# Anogenital Verrucous Carcinoma—A case report

**DOI:** 10.1016/j.ijscr.2018.11.017

**Published:** 2018-11-27

**Authors:** Hannah Trøstrup, Steen H. Matzen

**Affiliations:** Department of Plastic Surgery and Breast Surgery, Zealand University Hospital, Roskilde, Denmark

**Keywords:** Anogenital verrucous carcinoma, Squamous cell cancer variant, Case report

## Abstract

**Introduction:**

Verrucous carcinoma is an uncommon variant of low-grade squamous cell malignancy with a low malignant potential but a high risk of recurrence.

**Presentation of case:**

We report a case of a Human Papilloma Virus negative local verrucous carcinoma in the perianal area of a 45-year old otherwise healthy female. The tumor presented clinically as a persistent genital wart not responding to usual dermatologic local therapy. The patient was referred to the Department of Plastic Surgery and Breast Surgery after primary excision in a private practice setting of a general surgeon. Three months later, further excision revealed local lichen sclerosus inflammatory changes in close proximity to the excision scar.

**Discussion:**

Due to the low occurrence of verrucous carcinoma in the perianal area, no guidelines are available for the management of this disease. Conflicting reports on the ethiology and the nomenclature and classifications of verrucous carcinomas exist.

**Conclusion:**

Avoid pitfalls in the diagnosis of a rare variant of squamous cell cancer with a high recurrence ratio: Common genital warts not responding to ordinary local antimitotic Podophyllotoxin treatment require biopsy to exclude verrucous carcinoma. Follow-up is recommended at a 3–6 months interval due to the relative high risk of relapse.

## Highlights

• Verrucous carcinoma (VC) is a variant of squamous cell carcinoma.
• Anogenital VC is a rare condition, which clinically presents as common genital warts.
• Early recognition of VC and radical excision is crucial due to local destruction of tissue.
• Recurrence of VC is not uncommon.

## 1 Introduction

Verrucous carcinomas are rare, low-grade warty exophytic squamous cell carcinomas (SCC). Verrucous carcinomas were first described by Ackermann in 1948 in the oral pharynx. However, the lesions can also be located to the larynx or plantar surface of the foot (epithelioma cuniculatum or carcinoma cuniculatum). In the anogenital region, the tumor may present as condyloma accuminatum, common genital warts. Dawson et al. described for the first time anorectal giant condyloma accuminatum in 1965 [1]. A morfologically alike tumour in the anogenital area is the rare sexually transmitted disease Giant Condylomata of Buschke and Lowenstein tumour (BLT), which was first described in 1925 [2]. It is believed to account for 5–16% of all penile carcinomas [3]. It is not clear whether VC and BLT belong to the same entity of tumours or not.

Due to the low incidence, no estimates of cutaneous presentations of verrucous carcinomas of the anogenital area exists. It is more common in middle-aged white individuals, predominantly males [4] or in immunocompromised patients. In females, it is more often localised to the vulva, whereas perianal verrucous tumours are more seldom. Lichen sclerosis (LS) may predispose to verrucous carcinomas [5,6].

Histopathologically, the tumor grows locally in a papillary manner, destructing surrounding tissue, but distant metastases are uncommon. Pushing tumour margins as opposed to infiltrating margins as seen in SCC is described histopathologically. Early, radical excision is crucial.

## 2 Case

**Fig. 1:**
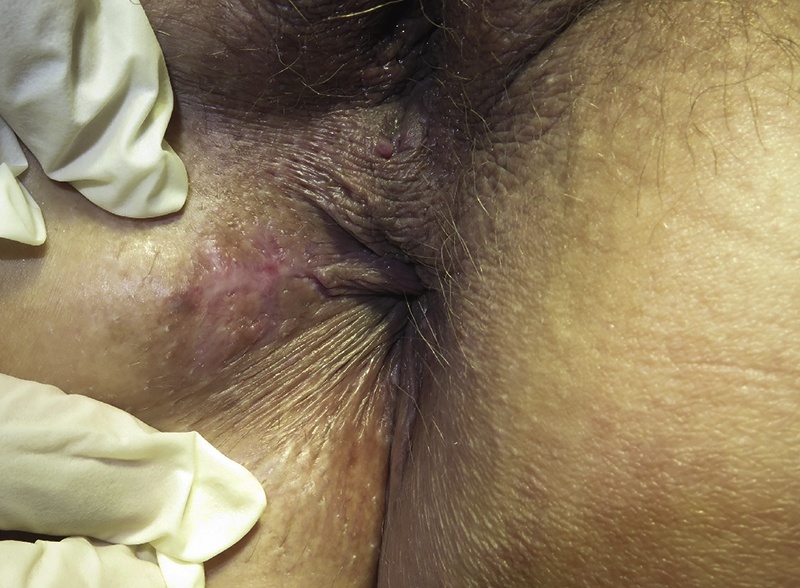
The postoperative appearance of the excised verrucous carcinoma: A mobile 20 × 18 mm scar, located at 7–9 o-clock in the perianal area is observed.

A 45- year old unmarried heterosexual, otherwise healthy and non-smoking female with no previous history of cancer, diabetes or lichen sclerosus was referred to the Department of Breast and Plastic Surgery for reexcision of a histopathologically verified 20 mm x 10 mm perianal verrucous carcinoma. The resection base margin was not free, so the patient was referred to Dep. of Plastic Surgery for further surgical intervention and follow-up. Histopathologically, there was some discussion whether the tumor excised prior to admission should be described as a verrucous carcinoma or a Buschke Loewenstein tumour. However, since no Human Papilloma Virus (HPV) could be found in the tissue by immunohistochemical staining, the final conclusion was verrucous carcinoma of the anogenital area (Fig. 1).

The tumour had been present and growing for 3 months. As local treatment by Podophyllotoxin failed to treat the lesion, the patient was referred to a general surgeon in a private clinic, who had excised the tumor. Resection borders were not reported.

No bleeding, itching, painful sensations or previous history of lichen sclerosus were reported. The patient had no risk factors for HIV or symptoms from the gastrointestinal tract. The only medicine prescribed was peroral Imigrane for intermittent migraine. No lymphadenopathy was found.

**Fig. 2:**
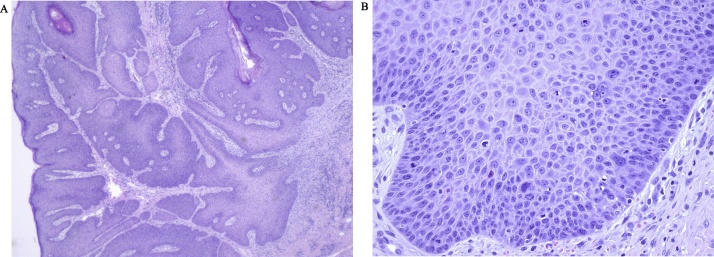
a) H&E stained slide (magnification ×2.5) of the verrucous carcinoma (primary excision). Epidermis is seen to the upper left. Explicit dyskeratotic changes are observed. b) H&E stained slide (magnification ×25) of the verrucous carcinoma (primary excision). Note the several mitosis in proximity to the basal membrane.

The clinical presentation was a scar (a) of 20 × 18 mm located in close proximity to the anus remained from the primary excision (Fig. 1). A stalked, soft mobile tumor of 5 × 4 mm (b) was also observed in the perianal region at 4 o’clock (Fig. 2). Histopathologic evaluation of a 4 mm punch biopsy from lesion b determined that is was a benign fibroepithelial tumor. The patient was referred to the department of surgical gastroenterology, Herlev, Denmark, for endoscopy and further surgical intervention on the suspicion of involvement of the anal canal. PET-CT scan determined no signs of dissemination. A transrectal ultrasound examination revealed a tumour observed at 4 o’clock. This tumour involved the internal muscular sphincter of the anus and it was excised in toto. Histologic diagnosis was lichenoid inflammation.

Follow-up was set for 6 months in which the patient did not display any signs of relapse (Table 1).

**Table 1:** Clinical conclusions.

*Management of verrucous carcinomas in the anogenital area:*
Verrucous carcinomas of the anogenital area are rare, low-grade squamous cell cancers clinically resembling common genital warts Meticulous surgery including subcutaneous tissue should ensure free surgical margins Long term follow-up due to the propensity for recurrence Long term prognosis is good Clinicians should consider verrucous carcinoma in case of failure of local antimitotic Podophyllotoxin treatment of genital warts

## 3 Discussion

Verrucous carcinomas of the anogenital area are rare. They are low-grade variants of squamous cell cancer presenting as common genital warts [7]. Pathophysiological ethiologies for anogenital verrucous carcinomas suspected are Human Papilloma Virus (HPV) type 6 and 11 [4]. Immunohistochemical staining showed no HPV in the excised tissue. The final histopathological diagnosis was VC. Tumour staging was …

Differentiation between VC and BLT is clinically and histopathologically challenging. Zidar and collegues recently suggested that anal verrucous carcinoma should be distinguished from BLT due to the lack of HPV [8]. This might also explain the lack of effect of local treatment by Podyphyllotoxin to treat VC/BLT [8]. Some pathologists equate BLT to verrucous carcinoma of the anogenital region [9], however, other authors classify VC as an intermediary lesion between condyloma acuminata and squamous cell carcinoma [10,11].

In this case, excision was done before admission to our department, which also sets limitations to the study. However, it is our belief that early recognition of VC is crucial in order to prevent late effects of such tumours. In larger VCs, endoscopy, Computed Tomography (CT) or Magnetic Resonance

Imaging (MRI) may locate extent of the lesion prior to surgical intervention. Rigorous excision with standard surgical margins and excision of subcutaneous fat is required as recurrence of verrucous carcinomas has a poor prognosis. Recurrence is not uncommon. Long term follow is thus suggested at 3–6 months interval due to the frequent transformation to usual type squamous carcinoma.

## 4 Conclusions

Clinicians should be aware of genital warts not responding to ordinary gold standard treatment and should consider tissue biopsies in these cases.

This work is reported in line with the SCARE criteria [12] and as well the PROCESS criteria [13].

## Conflicts of interest

None declared.

## Funding

No funding was received.

## Ethical approval

The institution (Zealand University Hospital) exempts the case report from ethical approval.

## Consent

Written consent from the patient was given prior to the data collection.

## Author contribution

Hannah Trøstrup: study consent, design, data collection, interpretation, writing of the paper.

Steen H. Matzen: design, interpretation, writing of the paper.

## Registration of research studies

Not applicable.

## Guarantor

Steen H. Matzen.

## Provenance and peer review

Not commissioned, externally peer reviewed.
